# Neuronal Oscillatory Signatures of Joint Attention and Intersubjectivity in Arrhythmic Coaction

**DOI:** 10.3389/fnhum.2021.767208

**Published:** 2021-11-05

**Authors:** Alexander Maÿe, Tiezhi Wang, Andreas K. Engel

**Affiliations:** Department of Neurophysiology and Pathophysiology, University Medical Center Hamburg-Eppendorf, Hamburg, Germany

**Keywords:** non-rhythmic interaction, self-perception, joint attention, social cognition, hyper-scanning, EEG

## Abstract

Hyper-brain studies analyze the brain activity of two or more individuals during some form of interaction. Several studies found signs of inter-subject brain activity coordination, such as power and phase synchronization or information flow. This hyper-brain coordination is frequently studied in paradigms which induce rhythms or even synchronization, e.g., by mirroring movements, turn-based activity in card or economic games, or joint music making. It is therefore interesting to figure out in how far coordinated brain activity may be induced by a rhythmicity in the task and/or the sensory feedback that the partners receive. We therefore studied the EEG brain activity of dyads in a task that required the smooth pursuit of a target and did not involve any extrinsic rhythms. Partners controlled orthogonal axes of the two-dimensional motion of an object that had to be kept on the target. Using several methods for analyzing hyper-brain coupling, we could not detect signs of coordinated brain activity. However, we found several brain regions in which the frequency-specific activity significantly correlated with the objective task performance, the subjective experience thereof, and of the collaboration. Activity in these regions has been linked to motor control, sensorimotor integration, executive control and emotional processing. Our results suggest that neural correlates of intersubjectivity encompass large parts of brain areas that are considered to be involved in sensorimotor control without necessarily coordinating their activity across agents.

## 1. Introduction

Hyper-scanning is a term that describes the simultaneous recording of brain activity from two or more people while they undergo some form of interaction, and it has developed into an important empirical method for research in social cognition. The approach can employ all major signal modalities that are used in brain research, i.e., EEG, MEG, fMRI, and fNIRS. A consistent finding in hyper-scanning studies is that the brain activities of the interacting partners are temporally coordinated. Depending on the kind of the interaction, this coordination has been observed on different timescales and in different brain areas, suggesting that it involves a multitude of cognitive functions.

There is a spectrum of opinions about what functional role coordinated inter-brain activity might play (Hamilton, 2020). As discussed in detail by Konvalinka and Roepstorff (2012), hyper-brain activity is considered as substrate for a functional coupling between individuals that is used to underpin representational concepts of social cognition as well as dynamic or enactive accounts. In representational concepts, inter-brain coupling is seen as an enabling mechanism for shared representations of the task, goals, actions and intentions (Sebanz et al., 2006; Vesper and Sebanz, 2016). Enactive accounts emphasize the importance of the circular dynamics between interacting agents for social cognition (Fuchs and Jaegher, 2009; De Jaegher et al., 2010). More recently it has been suggested that social cognition at least in part relies on action-effect contingencies that agents deploy to predict their own and other's actions (Maye and Engel, 2016). In this framework, hyper-brain activity would be seen as an indicator for the dynamical informational and sensorimotor coupling of agents in the interaction (Lübbert et al., 2021).

The paradigms that are used in hyper-scanning studies can be roughly separated into those in which the interaction happens in turns, like in card games, or is continuous, like imitating movements. The prisoner's dilemma game may serve as a representative example for a turn-based interaction paradigm. In each turn, the players decide whether to “cooperate,” “defect” or adopt a “tit-for-tat” strategy, and the combination of responses determines the reward received by the dyad. By quantifying information flow between the two brains during the period when partners make their decision by partial directed coherence, networks have been observed that change their topology depending on the combination of strategies the partners followed (Babiloni et al., 2007a; De Vico Fallani et al., 2010). An example paradigm for studying inter-brain coupling during continuous interaction is making music together. Guitarists showed increased inter-brain phase synchronization in the theta band at fronto-central electrodes when setting their tempo to the beats of a metronome and around the onset of playing a short melody together (Lindenberger et al., 2009; Sänger et al., 2012). After the onset however, this synchronization decreased. Since the reported inter-brain synchronization effects were all in the low-frequency bands, the authors suggest they may result from the similarity of the temporal properties of sensorimotor processes in the individuals.

One problem in hyper-scanning studies is to disentangle coherent inter-brain activity related to the interaction and merely correlated activity resulting from common input (Hamilton, 2020). Such correlated activity has been demonstrated between participants watching the same movie but not at the same time (Hasson et al., 2004), so that there clearly is no interaction between them. Another issue, in our view, is that the majority of tasks in hyper-scanning studies requires that the physical activity of the partners be temporally coordinated; therefore, the question arises in how far the observed inter-brain coupling can be traced back to the temporal coordination required by the task structure. In the aforementioned guitar players study, the authors suggest that the observed coupling of brain activity in the low frequency range is likely related to the partners coordinating their behavior through reciprocal sensorimotor feedback. Konvalinka and Roepstorff (2012) must have had a similar feeling when they wrote:

“Therefore, as mutual interaction involves behavioral coupling between two people producing similar actions, and engages similar cognitive processes (such as predicting each other's actions, imitating each other's hand/finger movements, and jointly attending to joint actions) between interacting partners, it may not be so surprising that their brain rhythms are synchronized.” (p. 7)

This begs the obvious question of how much and what kind of inter-brain coupling one would observe if the task would not impose strong temporal coordination of behavior. Corresponding studies are scarce though. The room cleaning scenario described in Dodel et al. (2010) may be one of the few examples. The task for the team is to enter a previously unseen room and “clean” it by keeping enemies who are potentially lingering in the room in check. Solving the task requires the team members coordinating their behavior on the tactical level without involving precise synchronization of the actions. In a hyper-scanning version of the paradigm in a virtual environment, the researchers found changes in the intrinsic dimensionality of brain activity with exercising the task, but they did not report any inter-brain coupling results (Dodel et al., 2011). Another study that observed the unfolding of coordinated room “cleaning” in a simulated environment (Tognoli et al., 2011a) reported candidate neuromarkers comprised of different EEG topographies and different frequency bands that characterize events during the action, but it also did not report any inter-brain coupling (Tognoli et al., 2011b).

To help fill this gap we investigated coupling of neuronal oscillatory activity in a joint target-tracking task. In this game of skill, two players had to adjust two orthogonal axes of a tablet in order to make a virtual ball roll toward a moving target and follow it as closely as possible. Each player minimized the distance between the ball and the target along his or her axis of control, but whether they hit the target at the same time or not was not relevant for the success. We show that the task induced oscillatory activity in sensory as well as motor areas of the brain, which is an obvious expectation if one looks for neuronal mechanisms of social interaction. We go one step further though and demonstrate that these activity patterns co-vary with the subjective experience of the interaction in terms of the own performance in the task as well as the success of the collaboration. We were particularly interested to figure out in how far properties of the inter-brain coupling would be related to the subjective experience. To address the problem of common input, we contrasted two conditions in which both players received the same input but worked on the task together or individually.

This article complements a previous report about the same dataset in which we analyzed physiological signals (heart beat, respiration, skin conductance). The analyses revealed that autonomic parameters during the game are predictive for the self-assessment of the own performance and the success of the collaboration to be rated after each trial (Maye et al., 2020). Taking this finding as an indication for the role of bodily processes in the emergence of intersubjectivity, we here aim to elucidate the contribution of activity in the brain to this experience.

## 2. Methods

### 2.1. Joint Target Tracking Task

The main objective in the search for an experimental paradigm was that it should make the partners continuously collaborate on a task but without the need for rhythmic temporal coordination. In order to be optimally sensitive for identifying underlying EEG dynamics of implicit interpersonal interaction, the second objective was to minimize artifacts induced by movements of the subjects.

**Figure 1:**
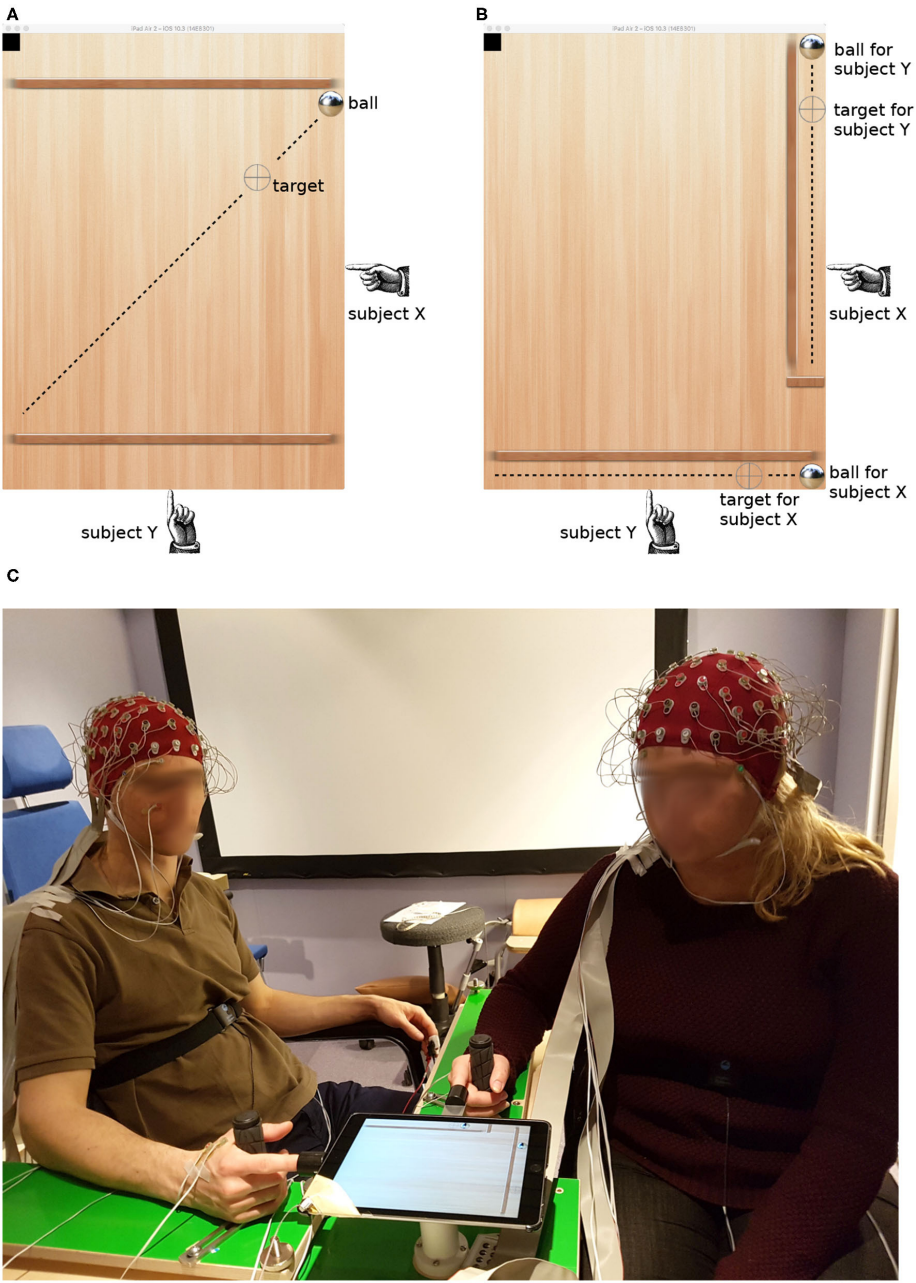
Target tracking task in the collaborative **(A)** and individual condition **(B)**. Dashed lines visualize the trajectory of the target; they were not visible to the subjects. The picture **(C)** shows the experimental setup and two participants with EEG caps.

We therefore decided to adopt a virtualized version of the common labyrinth game in which one or more players move a ball to a target location by tilting the game board. In order to keep the two players in continuous interaction, we employed a moving target that reversed its direction at random intervals (see Figure 1A). Players controlled the tilt angle of the board along orthogonal axes. This non-redundant control mode did not allow one player to compensate errors made by the other; instead, both players independently minimized the distance between the ball and the target along their control axis. The maximum distance the target moved in one direction was 14 cm, and it traveled at about 0.86 cm/s.

Dyads were instructed to keep the ball as precisely as possible on the target. We used the Euclidean distance *d* between the ball and the target, accumulated along the duration of a round (*T*), as an objective measure of performance:


$$d=\overset{T}{\underset{t=1}{\sum }}\sqrt{\Delta x^{2}+\Delta y^{2}},$$

where Δ*x* and Δ*y* are the distances along each player's axis.

We contrasted this collaborative condition with another configuration which was similar in its sensorimotor feedback but different in the level of interaction. In the individual condition, there were two balls and two targets, and each player tried to bring them together individually (see Figure 1B). Both targets still reversed their movement direction at random times; however, they did so at the same time. The reversal times were different in each round, thus minimizing the temporal coherence of common input across trials and dyads.

The paradigm was implemented on a tablet computer (iPad2, Apple Inc.). The kinematic of the virtual ball was driven by Newton's second law with the accelerations given by the tilt angle of the tablet. The tablet was mounted on a ball joint that carried its weight. Participants poked their index finger into a thimble on two sides, clenching the other fingers around a handle and resting their forearm on an L-shaped frame (see Figure 1C). This arrangement enabled the players to tilt the tablet by effortless, miniature movements of the index finger, minimizing artifacts in the EEG caused by muscle contractions.

### 2.2. Participants and Experimental Procedure

Twenty-eight subjects participated in the study (20 females, mean age 25.18 ± 3.86 years). All participants were right-handed and reported to be in healthy condition. Except for 2 dyads, all participants declared to not know each other in the first session. We obtained written informed consent before commencing the experiment, and the participants received financial compensation. The study was approved by the ethics committee of the medical association of the city of Hamburg.

Participants exercised the task for 6 consecutive days with the same partner. On each day, they completed 7 rounds in each condition (collaborative/individual) in random order. Each round lasted about 2 min. The training phase allowed the participants to develop a dynamically stable performance on the task and to acquire a routine for the experimental procedure. Hyper-scanning was carried out on days 7 and 8. On day 7, they played with their training partner; on day 8, they played with another subject from the cohort. The analysis did not reveal any significant differences between the data from days 7 and 8; therefore, and to support the statistical power of the analyses presented below, data from both days were pooled.

Immediately after each trial, the experimenter requested the participants to rate their feeling regarding the following aspects:

R1: “Please rate your own performance.”
R2: “Please rate your partner's performance.”
R3: “Please rate the collaboration.”

Participants selected a number between 1 and 9 (1-very poor, 9-excellent) on a small remote control which they held in their left hands underneath the armrest so that the partner could not see their selection. R2 and R3 were called out only after a collaborative trial. We consider responses to R1 and R2 as subjective measures of performance, whereas R3 reflected the success as a team.

### 2.3. Data Recording and Analysis

The experiment took place in an electromagnetically shielded and sound attenuated chamber. EEG was recorded simultaneously from 64 active electrodes on the scalp of each participant using two synchronized amplifiers (ActiveTwo AD-box, BioSemi instrumentation) with a sample rate of 2,048 Hz. Electrodes were placed according to the international 10/20 system and mounted in stock head caps from the same company. Electrode montage was standardized by centering Cz between nasion and inion and between the pre-auricular points.

Horizontal eye movements were recorded from two electrodes placed at the outer canthi. To record vertical eye movements, two more electrodes were mounted above and below the right eye of each participant (see subject on the left in Figure 1C). The horizontal and vertical components of the EOG were determined by subtracting the signals from the corresponding electrodes.

We also recorded physiological signals, i.e., ECG, respiration, EDA, and finger EMG. Details about the recording of those data and their analysis are given in Maye et al. (2020).

Data were analyzed in Matlab (The Mathworks, Natick, MA, USA) using the Fieldtrip toolbox (Oostenveld et al., 2011).

#### 2.3.1. Preprocessing

The recorded EEG data were segmented into epochs of 109.7 s aligned to the start of each round. A 0.5 s zero-padding was added at the beginning and the end respectively. The data were then re-referenced to the common average and filtered by a two-pass finite impulse response filter with Hamming window in the frequency band of 0.5–256 Hz. A notch filter was used to remove power line noise and its harmonics. Linear trends in the EEG data were also removed. The data were then resampled to 512 Hz.

In order to remove artifacts resulting from muscle activity, eye movements and blinks from the EEG data, we employed independent component analysis (ICA) over all 64 EEG channels. The artifact-typical components were manually identified, verified and removed. Artifactual components were identified by matching their patterns and time courses to those shown in Jung et al. (2000). In particular, artifacts resulting from eye movements and blinks, muscular activity on the scalp and in the neck and from cardiac activity were detected and removed. The EOG was not included in the ICA but was used for verifying the correctness of ICA. The time course of components with typical eye movement-related patterns were compared with the EOG to make sure all ocular artifact-related peaks were tracked by the ICA components with the typical patterns.

#### 2.3.2. Localizing EEG Sources

A three-dimensional current distribution was reconstructed from the signals of the 64 scalp electrodes by eLORETA (Low Resolution Electromagnetic Tomography, Pascual-Marqui et al. 1994). This method offers exact, zero error localization to point-test sources (Pascual-Marqui, 2007). The standard boundary element method (BEM) head model provided by the Fieldtrip toolbox was used (Oostenveld et al., 2003) with a voxel edge length of 1 cm. EEG data were filtered by the inverse solution, yielding an activity trace at each voxel. The spectra of these source activities were calculated by multi-taper FFT and correlated with the behavioral indicators using Pearson's coefficient of correlation.

#### 2.3.3. Coupling Analysis

Data were divided into overlapping windows of 2 s and 1.5 s overlap. Using 1-s-windows did not qualitatively change the results. Since our analyses are focused on oscillatory brain activity, we used multi-taper FFT to transform EEG data to the time-frequency domain. Tapers were calculated from discrete prolate spheroidal sequences (DPSS), and 3 tapers were used for calculating the complex spectrum for each data window (1,024 samples). The frequency range was 1–120 Hz in steps of 1 Hz.

Rather than calculating phase coherency across trials, we here were interested in the stability of phase differences across time, i.e., the duration of a game. To this end we calculated the auto-cross-spectra *S*_*X*_ and *S*_*Y*_, the hyper-cross-spectrum *S*_*XY*_, and calculated coherence *C* by:


$$C=\frac{|S_{XY}{|}^{2}}{S_{X}S_{Y}}$$

Using circular coherence (Burgess, 2013) did not qualitatively change the results.

We also calculated other hyper-brain coupling measures that are frequently used in the literature: amplitude or power correlation (AC/PC), phase-locking value (PLV, Lachaux et al., 1999), partial directed coherence (PDC, Baccalá and Sameshima, 2001; Baccalá et al., 2007), and directed transfer function (DTF, Kaminski and Blinowska, 1991). We used the standardized interface to these methods that is provided by the Fieldtrip toolbox.

#### 2.3.4. Statistical Evaluation

The main comparison is between the strength of the respective coupling measure in the collaborative and the individual condition. Contrasting coupling at the sensor level and in source space likewise involves a combinatorially large number of comparisons (Maris et al., 2007). We employed cluster-based permutation tests to counteract the multiple comparison problem. This non-parametric test has the capacity for incorporating biophysical constraints (Maris and Oostenveld, 2007), which here is that electrodes/voxels with differences in coupling between the conditions be compact and that similar differences be present also in nearby frequency bands. The main idea is to compare a test statistic for the condition difference with the distribution of the statistic when the comparison is made between data that have been randomly sampled from both conditions. We used 1,000 repetitions for this resampling process. For the test statistic, we employed a dependent samples *t*-test with a threshold of 0.05. As we did not have a hypothesis about the strength of coupling in either condition, we considered both tails in the permutation test.

In order to relate activity clusters to the literature, locations of maximum correlation were looked up in the brainnetome atlas^1^ (Fan et al., 2016) and the neurosynth database.^2^

## 3. Results

### 3.1. Correlations Between Ratings

**Figure 2:**
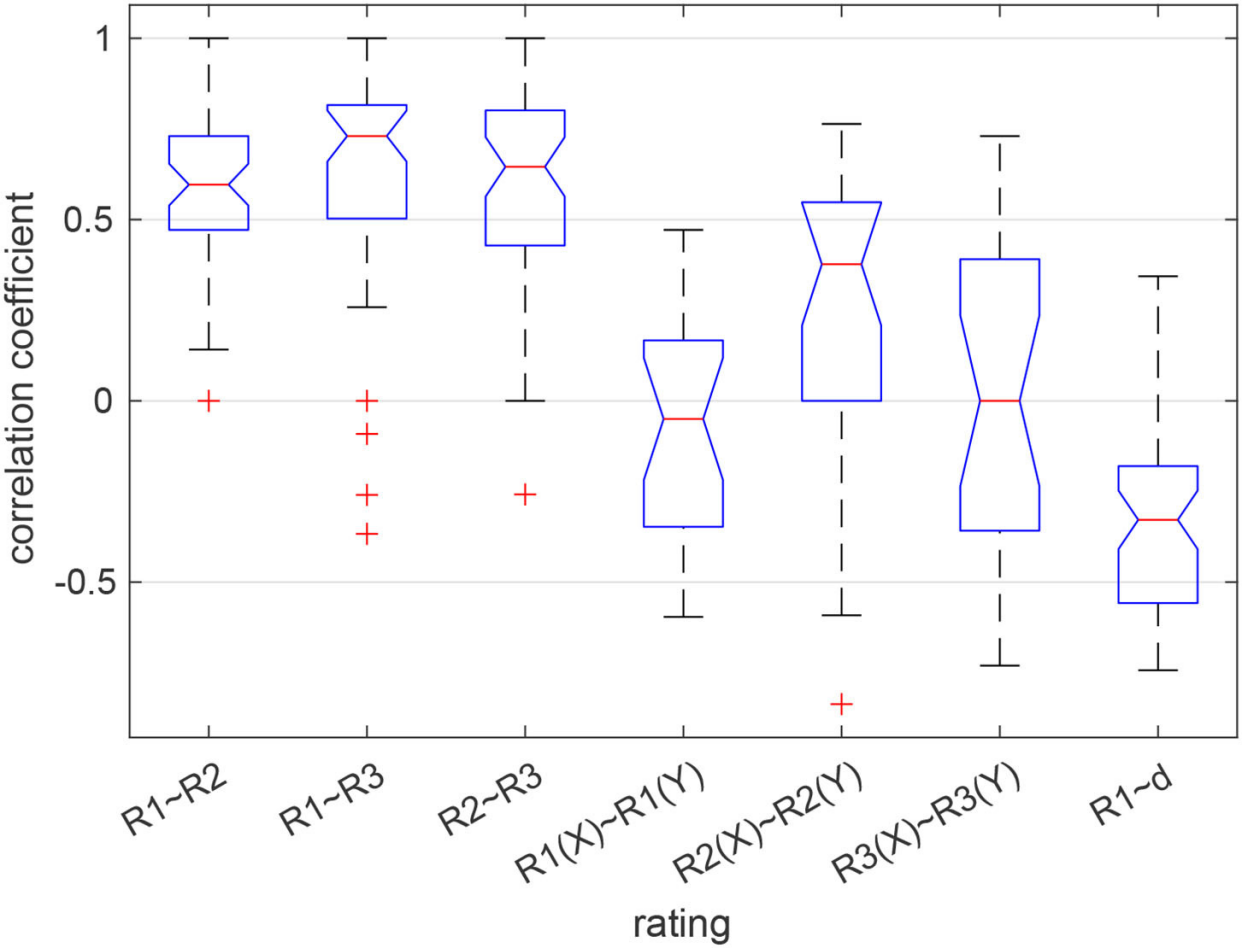
Correlation between the ratings given by each participant (3 boxes on the left) and between the partners in a dyad (next 3 boxes). The box on the right shows the distribution of correlations between the self-assessed performance (R1) and the time-accumulated distance between the ball and the target (d). Red lines show the median, blue boxes the 25 and 75% quantiles, whiskers the most extreme values not considered outliers, and red crosses show outliers. Medians are different at the 5% level if the notches do not overlap.

A previous analysis of the behavioral data revealed that there was no relation in how the two partners in a dyad evaluated their task performance with respect to the questions R1–R3. However, the three ratings were significantly correlated within individuals (Maye et al., 2020). Here we summarize this finding by showing the distribution of intra- and inter-individual correlations of the ratings in Figure 2. Whereas individual ratings were correlated with a coefficient of 0.6 or larger on average, the average inter-individual correlation of responses was around zero. Only when rating the partner's performance (R2), responses were correlated with a coefficient of about 0.4. The relation between the self-evaluation of the own performance (R1) and the tracking error (d) as an objective performance indicator was surprisingly weak (median correlation: −0.33), suggesting that participants did not accurately reflect upon their actual task performance.

### 3.2. Power Differences

**Figure 3:**
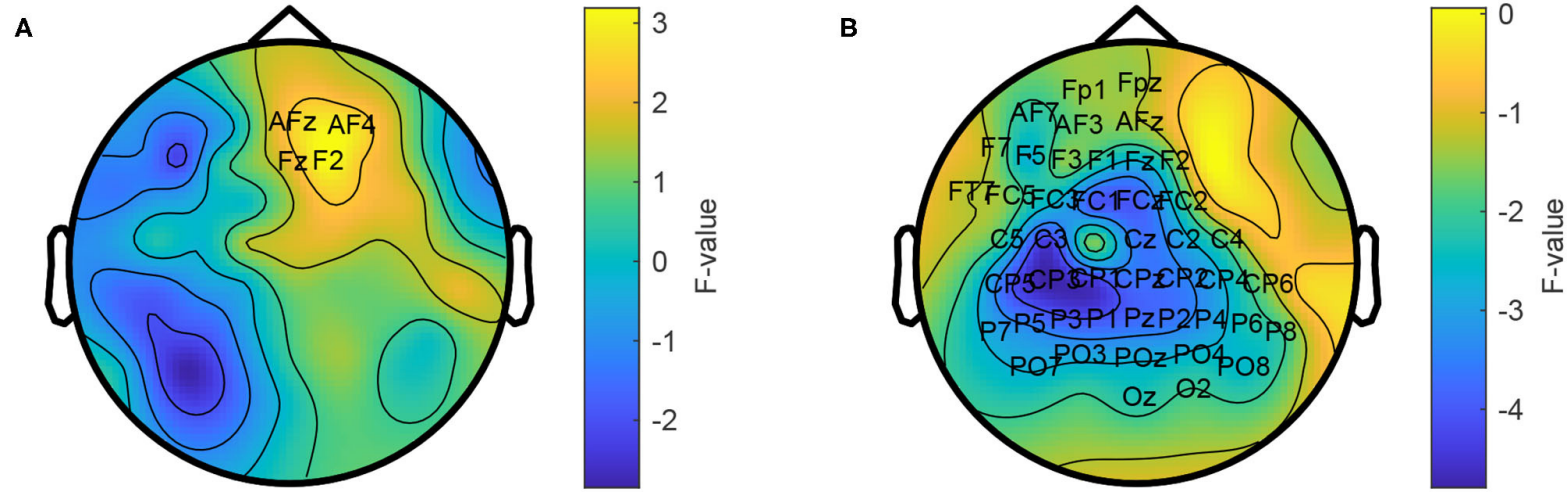
Topographic comparison of power spectra in the collaborative and individual condition. Power in the frequency range from 66 to 120 Hz is stronger in the collaborative condition **(A)** and weaker in the range from 1 to 30 Hz **(B)**. The average power across the respective frequency ranges is shown. Labeled electrodes indicate statistically significant differences.

We calculated the power spectrum for each trial and compared it between the two conditions. Across all participants, power in the range from 66 to 120 Hz was larger in the collaborative condition at a small group of fronto-right-central electrodes (p = 0.015, see Figure 3). The maximum difference was observed at electrode F2 at 113 Hz (F = 4.0).

In addition, the individual condition showed a power increase in the range from 1 to 30 Hz at a left-temporo-central region (p = 1e-3). The maximum difference was observed at electrode Pz at 14 Hz (F = 8.79).

**Figure 4:**
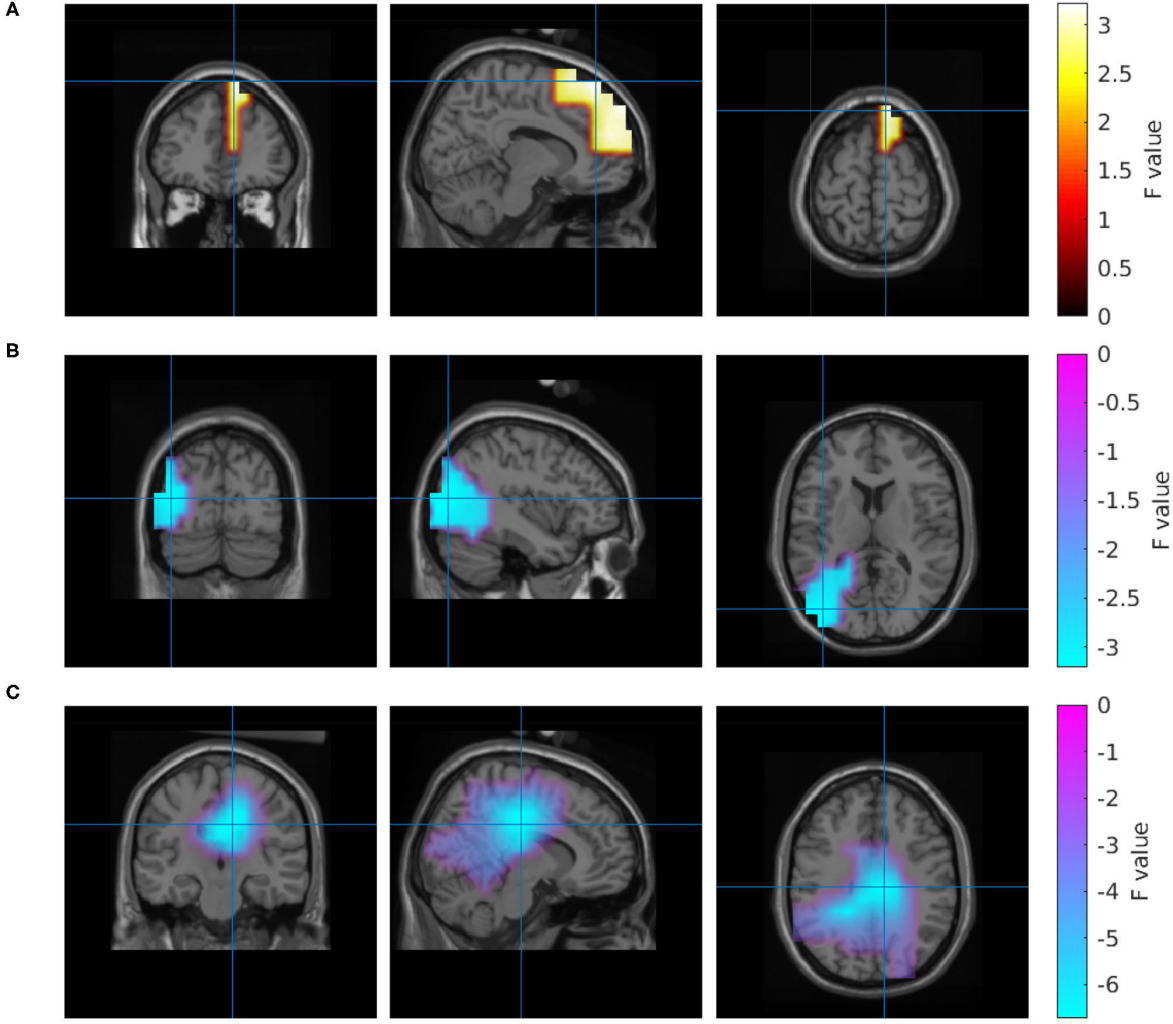
Source reconstruction of the power differences between the collaborative and the individual condition in the frequency range 66–120 Hz **(A,B)** and 1–30 Hz **(C)**.

To better understand the origin of these differences, we calculated an inverse solution in the brain's 3D source space. According to this solution, the power increase in the high gamma band during the collaborative condition was located in the right superior frontal gyrus (Figure 4A). The power decrease in the 1–30 Hz band emerged from the right cingulate gyrus (Figure 4C). The source reconstruction revealed another cluster with reduced activity in the 66–120 Hz band in the collaborative condition which was located in the left lateral occipital cortex (Figure 4B). A projection of this decrease can be seen at left parietal electrodes around P5 in the topography (Figure 3A); however, the statistics of this condition difference was above threshold there. Table 1 summarizes the frequencies, locations, and statistics of the power difference sources.

**Table 1:** Contrasting brain activity in the collaborative and individual conditions.

**Freq. (Hz)**	**Region of maximal correlation**	**MNI**			***p*-value**	**F statistic**
66–120	Right superior frontal gyrus A9l, lateral area 9	10	40	60	0.033	3.23
66–120	Left lateral occipital cortex mOccG, middle occipital gyrus	−40	−80	10	0.033	−3.21
1–30	Right cingulate gyrus A23v, ventral area 23	10	−20	40	0.001	−6.76

### 3.3. Hyper-Brain Coupling

In order to search for signs of coordinated brain activity in the dyads, we analyzed the EEG data from the partners using the following coupling methods: amplitude coupling (AC), power envelope coupling (PC), partial directed coherence (PDC), directed transfer function (DTF), Granger causality (GC), coherence (COH), and phase-locking value (PLV). None of the methods indicated a systematic increase or decrease of coupling between the two conditions. The *p*-values of the respective cluster-based randomization statistics are listed in Table 2.

**Table 2:** Contrasting the collaborative and individual conditions using different coupling methods.

**Connectivity measure**	**Collab>indiv**	**Indiv>collab**
AC	0.13	0.51
PC	0.3	0.87
PDC	0.6	0.54
DTF	0.71	0.73
GC	0.07	–
COH	0.3067	0.2947
PLV	0.1948	0.3776

*p-values result from a cluster-based randomization test*.

### 3.4. Correlating Behavioral Data and Source Activity

We finally analyzed possible relations between the individual brain activity and the respective behavioral parameters of the participant. To this end, we reconstructed the activity in 3D source space and transformed it to the frequency domain. We then correlated the power spectrum at each voxel with the tracking error along the axis of the participant, motion energy and the three ratings R1–3.

**Figure 5:**
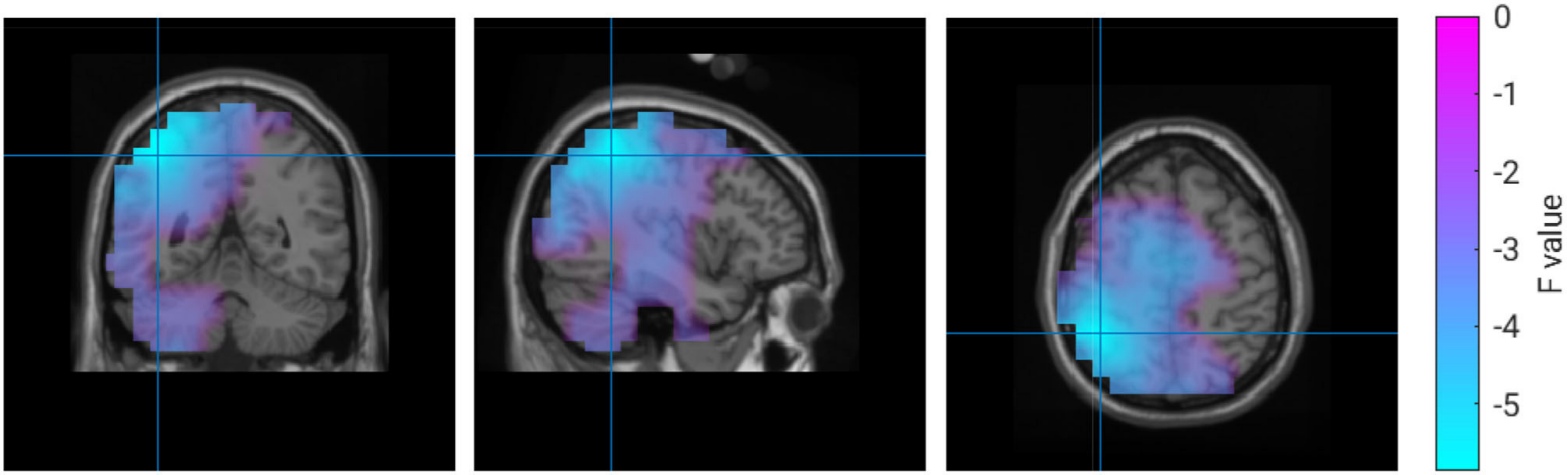
Correlation between 18 Hz power and tracking error.

We found a negative correlation between the tracking error and beta-band power in a region in the left inferior parietal lobule. Properties of this correlation are listed in Table 3, and a visualization of this region is shown in Figure 5.

**Table 3:** Clusters of brain activity correlating with tracking error.

**Freq**.	**Region of maximal correlation**	**MNI**	**Corr. coeff**.	***p*-value**	**F statistic**
18 Hz	Left inferior parietal lobule A40rd, rostrodorsal area 40 (PFt)	−40 −50 50	−0.21	0.001	−5.9151

**Figure 6:**
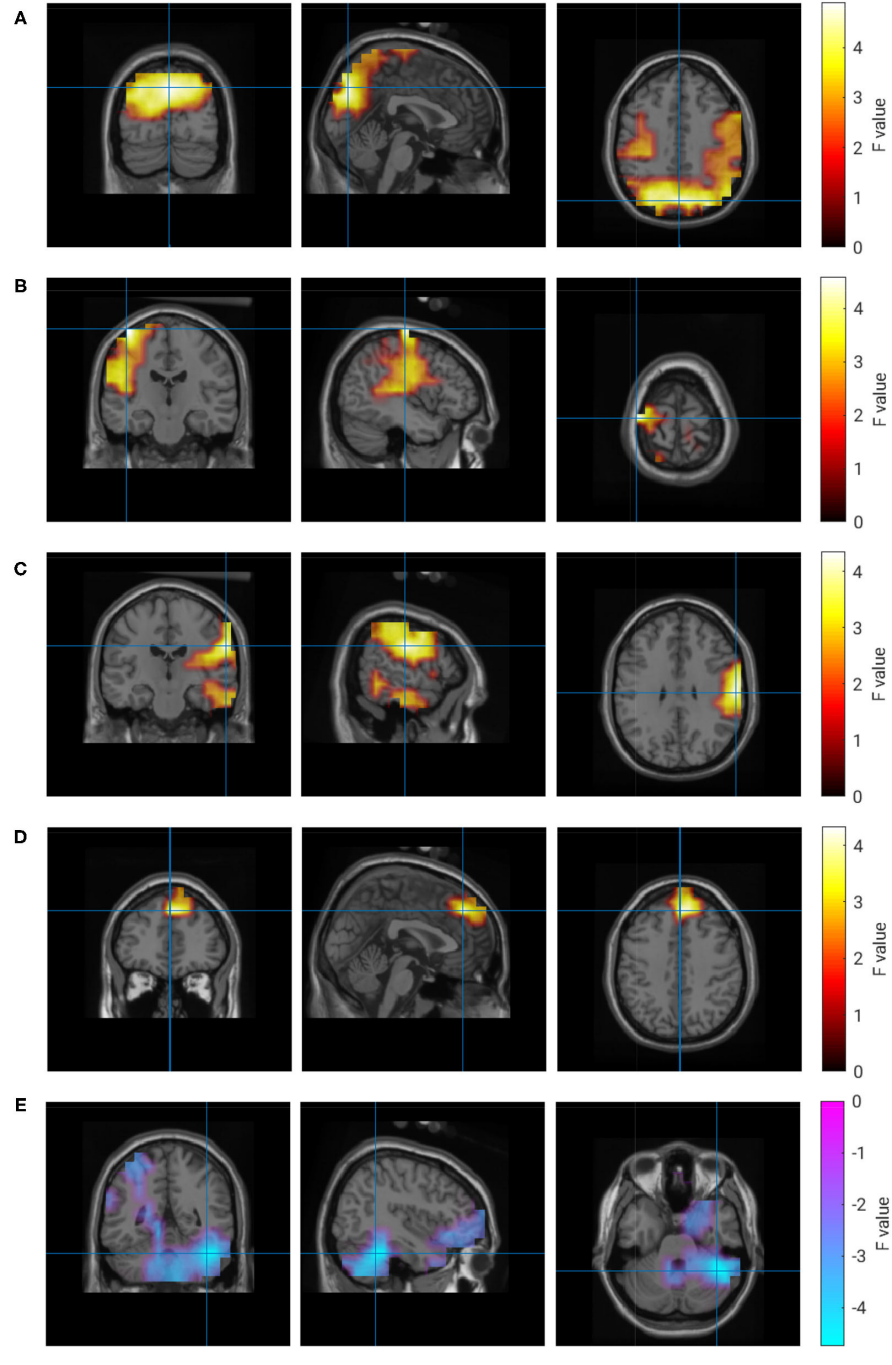
Correlation of brain activity and rating of own performance. **(A)** 10 Hz, **(B)** 25 Hz, **(C)** 22 Hz, **(D)** 22 Hz, **(E)** 1 Hz.

For the subjective experience of own performance (R1), the analysis revealed a more complex pattern of regions with correlated brain activity. A small frontal region, a larger occipital region and two left and right temporal regions comprise the set of brain regions that were positively correlated with ratings of the own performance. Whereas the strongest correlation in the occipital regions was observed in the alpha band, the remaining regions had their maximum correlation in the beta band. In addition, a negative correlation was detected in the right hemisphere of the cerebellum in the delta frequency range. Properties of these clusters are listed in Table 4, and Figure 6 visualizes their location and extension.

**Table 4:** Clusters of brain activity correlating with rating of own performance.

**Freq. (Hz)**	**Region of maximal correlation**	**MNI**			**Corr. coeff**.	***p*-value**	**F statistic**
10	Right precuneusA7m, medial area 7 (PEp)	0	−80	40	0.18	0.007	4.99
25	Left precentral gyrus A4ul, area 4 (upper limb region)	−40	−20	70	0.17	0.023	4.64
22	Right inferior parietal lobule A40rd, rostrodorsal area 40 (PFt)	60	−20	30	0.16	0.042	4.48
22	Right superior frontal gyrus A9m, medial area 9	0	40	40	0.16	0.042	4.48
1	Cerebellum	40	−50	−30	−0.17	0.011	−4.86

**Figure 7:**
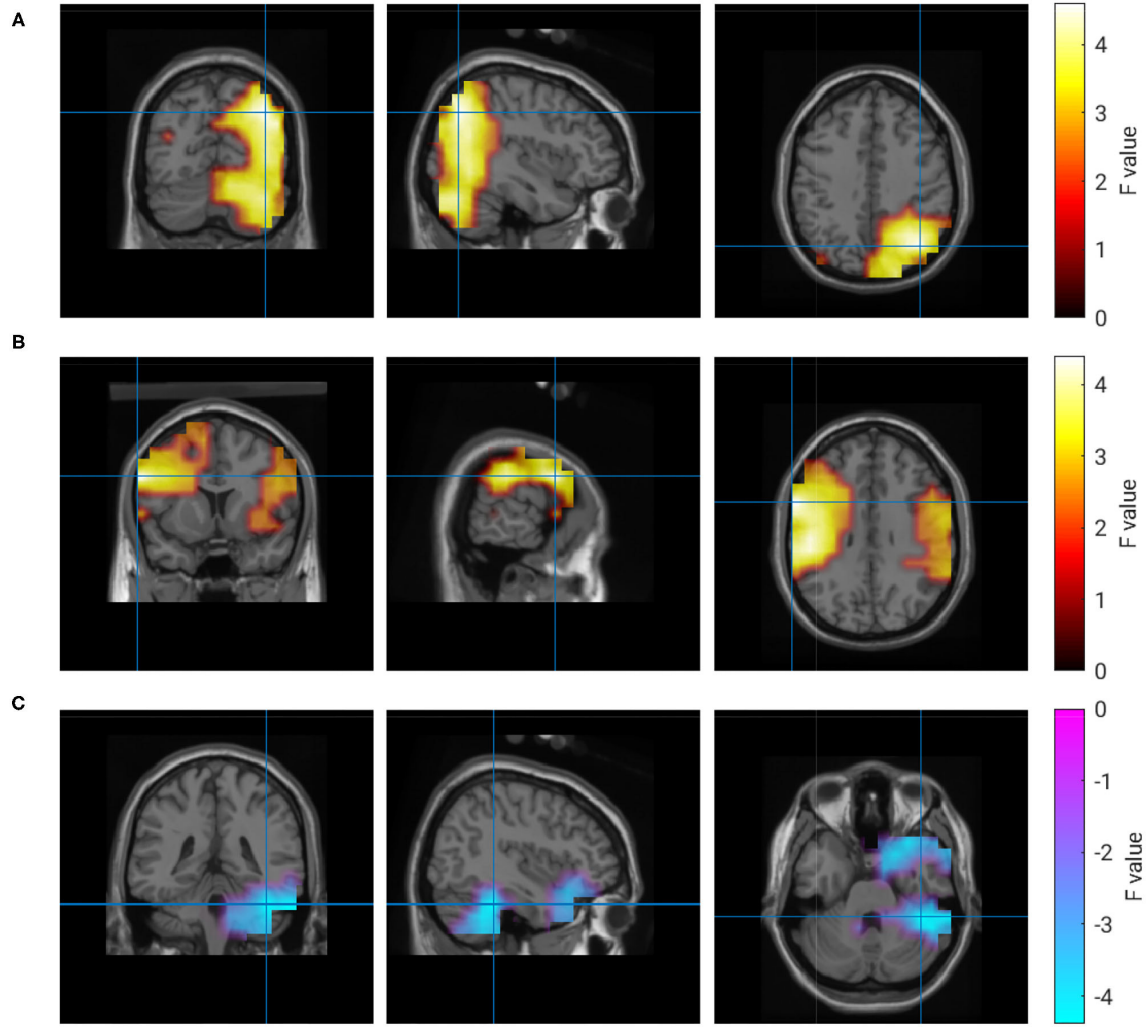
Correlation of brain activity and rating of the partner's performance. **(A)** 9 Hz, **(B)** 20–120 Hz, **(C)** 1 Hz.

The activity in four regions correlated with the ratings of collaboration (R3). Alpha-band activity in a right parieto-occipital region showed the maximum correlation. Similar to ratings of own performance, the experience of collaboration also correlated with activity in left and right temporal regions. In contrast to all other clusters, this correlation was not frequency-specific and could be observed in the range from 20 to 120 Hz. Again, a negative correlation at delta frequencies was found in the right hemisphere of the cerebellum. A quantitative description of the correlations with R3 is given in Table 5, and the regions are visualized in Figure 7.

**Table 5:** Clusters of brain activity correlating with rating of collaboration.

**Freq. (Hz)**	**Region of maximal correlation**	**MNI**			**Corr. coeff**.	***p*-value**	**F statistic**
9	Right inferior parietal lobule A39rv, rostroventral area 39	40	−70	740	0.23	0.02	4.66
20–120	Left precentral gyrus A4hf, area 4 (head and face region)	−60	10	30	0.22	0.036	4.49
1	Cerebellum	40	−40	−30	−0.22	0.058	−4.45

There were no correlations with motion energy (*p* > 0.14) or ratings of the partner's performance performance (*p* > 0.1) found.

## 4. Discussion

### 4.1. Joint Action or Joint Attention?

The joint target tracking task required the two players to coordinate their actions in space and time and therefore complies with a working definition of joint action (Sebanz et al., 2006). It has to be pointed out though that the non-redundant control of the ball along orthogonal axes completely decoupled both agents' action and effect spaces. A feature which in our view is crucial in joint action, the adjustment of actions to those of the other agents, may therefore be missing in this paradigm. Hence interpretations of the results on the background of joint action should be taken with a grain of salt. Moving the virtual ball together with the partner in the collaborative condition and controlling it by oneself in the individual condition clearly should have switched between joint attention in the former and individual attention in the latter. Contrasting both conditions therefore can shed light on the neural processes involved in joint attention as a preliminary stage in joint action (Maye et al., 2017).

### 4.2. (No) Hyper-Brain Coupling in Arrhythmic Interaction

We applied a set of coupling analysis methods which have been used in the literature to reveal temporal coordination in hyper-activity data. None of them detected changes in hyper-brain coupling when partners switched between solving the task on their own and solving it together. One explanation for this apparently disappointing finding may be that the manipulation of the social context was just not strong enough to detect hyper-brain coupling. We think it is difficult to explain then, however, why switching between joint and individual target tracking should entail weaker changes in social coupling than, for example, switching strategies (cooperation/competition, collaborating/defecting) in card or economic games, e.g., (Babiloni et al., 2007b; De Vico Fallani et al., 2010), or in sports games (Liu et al., 2021). An alternative explanation could be that our paradigm did not impose rhythms which could be modulated by social context. It seems that some researchers also considered this possibility in their studies. For example, in Lindenberger et al. (2009) the authors discuss, that “. given that the reported rhythms were all in the low EEG frequency range, one plausible explanation could be that the similarities in sensorimotor feedback (at least partially) contributed to the inter-brain synchronization” (Konvalinka and Roepstorff, 2012). Thus, if it would not have been the similarity of the sensorimotor feedback as such but its rhythmic components^3^, then a lack of rhythmicity in the sensory feedback may explain our difficulties to observe modulations of the hyper-brain synchronization in the EEG in our paradigm. A recent study of hyper-brain activity in a virtual tennis game may round up this conclusion. Amplitudes of alpha- and beta-band oscillations were correlated when players were in the same team playing doubles (cooperative condition), but they were anti-correlated when playing in opposing teams (competitive condition) (Liu et al., 2021). Although the authors acknowledge the possibility that the observed coupling could be a by-product of the interaction in the game, they argue that the manipulation of the social context should prevail. Hence, a closer analysis of the effect of the interaction dynamics on the results in hyper-brain studies in general seems advisable.

### 4.3. Individual-Brain Signatures of Collaboration

More support for a successful manipulation of social context comes from the observation that tracking the target together or individually very well induced changes of the neuronal activities in the individual brains. When participants collaborated, we found increased high-gamma-band activity of a region in the right superior gyrus, which has been linked to explicit emotional processing (Iaria et al., 2008). At the same time, these oscillations were reduced in the lateral occipital cortex. Gamma oscillations in this region have been attributed to visual object processing, and they were modulated by attention and expectation (Tallon-Baudry et al., 2004). In addition, oscillations in the beta and lower frequency bands were also reduced under the collaborative condition in the cingulate gyrus, which likewise has been shown to be involved in visuomotor integration (Field et al., 2015) and social emotions (Britton et al., 2006). The topography of this decrease is similar to the result from a previous study on joint attention (Lachat et al., 2012), where the maximal modulation appeared between 11 and 13 Hz. We conclude that the two modes of solving the tracking task induced differences in visuomotor integration processes, emotional processing, and attention.

### 4.4. Neural Correlates of Task Performance and Self-Assessment

We also found several patterns of brain activity which were related to task performance and subjective experience. The tracking error showed a negative correlation with activity in the left inferior parietal lobule, i.e., stronger activity in this area was associated with better task performance. Activity in this region has been found for target motion prediction (Kawawaki et al., 2006), action execution, observation and imagination (Lacourse et al., 2005; Molinari et al., 2012; Jiang et al., 2015), linking it to the human mirror system (Dinstein et al., 2007). The correlation with task performance was specifically with activity in the beta band. This frequency band has been traditionally regarded as an idling rhythm in the motor system (Pfurtscheller et al., 1996), but newer accounts confer it a more active role for the maintenance of steady-state force output and a more efficient processing of proprioceptive feedback needed for monitoring the status quo and recalibrating the sensorimotor system (Engel and Fries, 2010). It may therefore well exhibit the “active akinetic process” that controls the miniature movements of the index finger to keep the ball on the target. Since beta rhythms are also related to the expectation of upcoming events, possibly they may also have been induced by the players waiting for the target to reverse its movement direction.

Whereas our analysis approach yielded a single activity cluster which correlated with the objective task performance, it revealed more complex spatio-spectral structures for the self-assessment of task performance. Taking into account the weak agreement between behavioral indicators of objective and subjective performance, i.e., tracking error and rating of own performance, this distinctiveness may come as no surprise. The maximum correlation occurred in the precuneus, a brain area which interestingly has shown activity for reflective self-awareness (Kjaer et al., 2002) and representation of the mental self (Lou et al., 2004; Cavanna and Trimble, 2006). But the cluster of significant correlation with ratings of task performance extends over large parts of the superior parietal lobule, which is considered a node in the default mode network. Interestingly, activity specifically in the alpha band, like in our study, showed significant overlap with the default mode network for self-referential thoughts and during a social game task (Knyazev et al., 2011). The researchers hypothesized that synchronization of internal mental processes as opposed to the processing of external stimuli might be the primary function of alpha oscillations in this region.

We found three additional clusters in which beta-band activity correlated with the outcome of the self-evaluation. One of these extended over the left motor cortex and therefore is likely related to movements of the right index finger. Activity in the inferior parietal lobule has been linked to motor representations of finger movements (Gerardin et al., 2003) and, as part of the human mirror neuron system, action observation and execution (Arnstein et al., 2011). The frontal activation cluster seems to match with the anteromedial portion of the right superior frontal gyrus, a region which again is part of the default mode network as well as the cognitive control network (Li et al., 2013). It may be interesting to note that the same region showed a stronger activation in the 66–120 Hz band during collaboration, whereas the correlation with self-assessment was found only for activity around 22 Hz. This may result from different neuronal populations with different activity profiles located in the same region or from the same population exhibiting a functional segregation by different frequency bands. In any case this comes as a reminder that the spatial and spectral activation profiles should be seen in an integrated fashion.

Whereas all clusters discussed so far were positively correlated with the self-assessment of performance, a region with negative correlation was located in the cerebellum. Traditionally, the cerebellum has been considered a site where models of the motor apparatus reside, and which are used for predicting the consequences of actions (Wolpert et al., 1998). But the cerebellum is also engaged in the acquisition and discrimination of sensory information (Gao et al., 1996), sensorimotor coordination, prediction and error-based learning, and affective socio-cognitive processing (Sokolov et al., 2017). With respect to tracking the target by finger movements in our paradigm, we think the cerebellar cluster can be closely linked with other studies which found that executed as well as imagined hand movements cause activity in the cerebellum (Lacourse et al., 2005), and that motor activity of and sensory signals from the fingers are mapped in the cerebellum (Wiestler et al., 2011). This link is further supported by the finding that the delta-band EEG has information which can be used to decode finger movements (Paek et al., 2014). Recently cerebellar activity has been linked to social cognition (Van Overwalle et al., 2020), and the correlation with the subjective performance evaluation could result from the observed goal-directed body movements of the partner in the context of our paradigm.

### 4.5. Neural Correlates of Intersubjectivity

Like for own performance, the experience of the success of collaborating with the partner was also correlated with activity in the left precentral gyrus. The extension of this cluster in the right hemisphere is very similar to the cluster around the right IPL that correlated with ratings of own performance. Whereas the spatial distribution of these clusters is similar between the two ratings, they differ in their frequency specificity. For ratings of collaboration, correlations can be observed in the beta band as well as across all of the gamma range, but for ratings of own performance, the correlation is specifically in the beta band.

The involvement of cerebellar activity also resembles the correlation structure for performance ratings. Recalling that ratings of own performance and collaboration were highly correlated, it would be interesting to know whether this was the result of the similarities in the neuronal activation profiles or whether our method yielded similar results because the input, i.e., the ratings, were correlated. Unfortunately it is not possible to answer this question with the approach selected for this study.

Despite these similarities, the spatial distribution of the alpha-band cluster exhibits notable differences between the two ratings. For ratings of collaboration, correlations were found only in the posterior part of the parietal lobe of the right hemisphere, extending into the ipsilateral occipital lobe, whereas it extends over both hemispheres for ratings of own performance.

The absence of correlations with motion energy and ratings of the partner's performance suggests two conclusions: First, the correlations with the other two ratings are not simply the result of how much the participants moved the tablet, at least not to a significant extent. And second, the evaluation of the partner's performance did not systematically covary with activity in the brain regions for motor control, sensorimotor integration and emotional processing of the own body like it did for evaluating the own performance or collaboration. This is insofar surprising as all three ratings were significantly correlated within the individuals. It is unlikely that the ratings were randomly given either, because they showed a stronger correlation within the dyad than the other two. We therefore conjecture a form of coupling of the partners in mutually rating their performance which our analysis methods were not able to pick up.

### 4.6. Action and Subjective Experience May Share the Same Neuronal Processes

Seen from a bird's eye perspective our analyses revealed several clusters in brain regions that are known to be involved in motor control, sensory processing, sensorimotor integration, and executive control. If one accepts that the observed correlations indicate, at least in parts, a causal relation, then the conclusion is that self-assessment of performance and collaboration are significantly modulated by the neuronal processes that govern sensorimotor coordination during the target-tracking task. This interpretation is supported by the finding that in the majority of clusters, oscillatory activity specifically in the alpha and beta band correlates with subjective experience. From the range of functional significance assigned to the two frequency bands, there is one aspect that sees alpha and beta oscillations together controlling task performance: motor inhibition. Following the target not only requires tilting the tablet in the right direction, but also involves suppressing unwanted movements and a great deal of precision in the motor control, both of which is not possible without inhibition. Stronger alpha oscillations may indicate better inhibitory control and tighter timing of cortical processing (Klimesch et al., 2007). Likewise, beta oscillations may be related to the maintenance of the sensorimotor set and the suppression of unexpected external events (Engel and Fries, 2010). It may also be hypothesized that the clustered activity in the alpha, beta and high gamma band are an index for cognitive operations of the global neuronal workspace (Palva and Palva, 2007); however, this would require showing phase coordination of these oscillations.

It has to be pointed out that the discussed brain regions have shown activity in many other tasks and contexts, and we selected the studies we deemed the most related to the experimental paradigm we investigated here. Nevertheless, the activation patters seem to match well with the cognitive requirements for solving the task. Together with the finding that the clusters were associated with specific frequency bands, the alternative interpretation of the observed correlations as sheer covariation seems less likely. Our analyses therefore support the view that the subjective experience of social interaction involves the interaction of distributed neuronal populations, many of which are considered controlling motor execution and coordinating sensorimotor processing. What's more, physiological processes in the body as indexed by autonomic parameters like heart rate variability, skin conductance and breathing rhythm also have been shown to be informative about experience of performance and collaboration (Maye et al., 2020). An integrated analysis of activity in the cerebral and autonomic nervous system, though extremely complex, may be a necessary next step toward a deeper understanding of the body for the emergence of intersubjectivity.

## Data Availability Statement

The raw data supporting the conclusions of this article will be made available by the authors, without undue reservation.

## Ethics Statement

The studies involving human participants were reviewed and approved by the Ethics Committee of the Medical Association of the City of Hamburg. The patients/participants provided their written informed consent to participate in this study.

## Author Contributions

The experiments were conceived and designed by AE, Mircea Stoica (see section Acknowledgments), and AM. The experiments were performed by Mircea Stoica and AM. The data were analyzed by AM and TW. The paper was written by AM, TW, and AE. All authors contributed to the article and approved the submitted version.

## Funding

The work described in this paper was supported by the EU through project ‘socSMCs' (H2020 – 641321) and by the DFG through project TRR 169/B1.

## Conflict of Interest

The authors declare that the research was conducted in the absence of any commercial or financial relationships that could be construed as a potential conflict of interest.

## Publisher's Note

All claims expressed in this article are solely those of the authors and do not necessarily represent those of their affiliated organizations, or those of the publisher, the editors and the reviewers. Any product that may be evaluated in this article, or claim that may be made by its manufacturer, is not guaranteed or endorsed by the publisher.

## Footnotes

^1^ atlas.brainnetome.org

^2^ neurosynth.org

^3^The study (Lindenberger et al., 2009) investigated guitarists playing a short melody together.
